# Pulmonary sarcoidosis with pleural involvement in a 44-year-old patient: a case report

**DOI:** 10.3389/fmed.2026.1786968

**Published:** 2026-03-16

**Authors:** Bin Zhang, Nailiang Zhai, Chengpeng He, Yongfu Xia

**Affiliations:** Department of Respiratory and Critical Care Medicine, Binzhou Medical University Hospital, Binzhou Shandong, China

**Keywords:** non-caseating granulomas, pleural effusion, pleural nodule, sarcoidosis, thoracoscopy

## Abstract

Although sarcoidosis is a granulomatous disease with unknown etiology involving multisystems, the pleura is rarely affected in this disease. Here, a case of sarcoidosis with pleural nodules and pleural effusion was presented. The patient suffered from dry cough and dyspnea lasting for 4 weeks. Chest computed tomography showed pulmonary nodules, subpleural nodules, mediastinal lymphadenopathy and bilateral pleural effusion. Thoracoscopic pleural biopsy was performed, and histopathological examination demonstrated non-caseating granulomas, confirming sarcoidosis-related pleural involvement. The patient was treated with corticosteroids, and all discomfort symptoms gradually regressed. A follow-up CT performed 8 weeks later exhibited the complete resolution of pleural effusion. The present case report highlights that when pleural effusion and pleural nodule are observed in a patient, sarcoidosis should be considered as the underlying disease while other potential causes are ruled out.

## Introduction

Sarcoidosis is an idiopathic granulomatous disorder that affects multiple organ systems, predominantly involving the lungs and lymphatic system, with the eyes and skin being the next most commonly affected sites (1, 2). Although pulmonary involvement is observed in over 90% of sarcoidosis cases, pleural involvement is rare. Reports indicate that the prevalence of pleural sarcoidosis is less than 3%, and the incidence of pleural effusion is merely approximately 1% (3, 4). The main manifestations of pleural involvement in sarcoidosis include pleural effusion, pleural thickening, and pleural nodules (5–7). Herein, we present a case of sarcoidosis complicated by both pleural nodules and pleural effusion, aiming to share relevant clinical practice experience.

## Case report

A 44-year-old Chinese female was admitted to hospital with a 4-week history of dry cough and dyspnea. She denied fever, fatigue, or unintentional weight loss. The patient worked as a bank clerk, with no history of exposure to inorganic particulate matter. There was no family history of autoimmune or other genetic diseases, and she had no prior medical history or smoking history. She had received empirical antibiotic therapy previously, which failed to alleviate her symptoms. Given these clinical manifestations, she was admitted to the Department of Respiratory Medicine for further management. On admission, her vital signs were as follows: body temperature 36.2 °C, pulse rate 93 beats per minute, respiratory rate 24 breaths per minute, and blood pressure 118/80 mmHg. Lung auscultation revealed no crackles bilaterally. The rest of the physical examination was unremarkable, with no skin nodules or lymphadenopathy noted.

Routine blood tests, coagulation function, and liver and kidney function were normal. Procalcitonin was 0.095 ng/ml (reference range: 0–0.046 ng/ml), while C-reactive protein (CRP) was not elevated. Serum carcinoembryonic antigen (CEA) was within the normal range. Several etiological tests, including the G test, GM test, and tuberculosis T-Spot test, yielded negative results.

**Figure 1:**
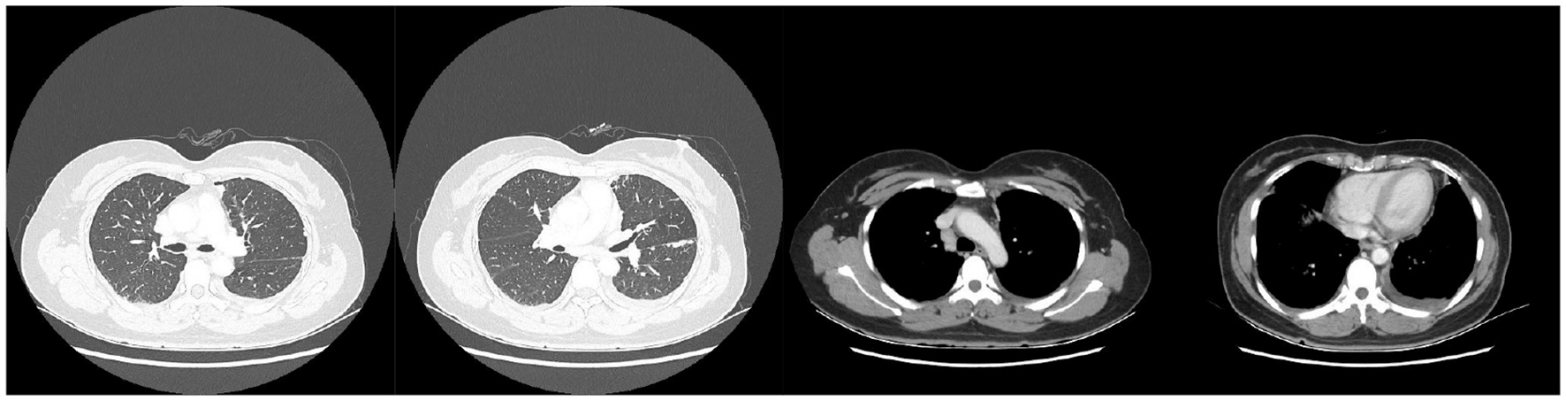
Chest CT showed pulmonary nodules, subpleural nodules, mediastinal lymphadenopathy and bilateral pleural effusion.

Chest computed tomography (CT) revealed pulmonary nodules, subpleural nodules, mediastinal lymphadenopathy and bilateral pleural effusion (Figure 1). Thoracentesis was performed, and the aspirated fluid was confirmed as exudate with a lymphocytic predominance (85%). The pathology of the pleural effusion revealed a large number of lymphocytes, with no malignant tumor cells identified. The lymphocyte count in the pleural effusion is approximately 2,200 cells/μl. The pleural fluid biochemical parameters were as follows: total protein 51.6 g/L, lactate dehydrogenase (LDH) 159 U/L, glucose 5.76 mmol/L, and adenosine deaminase (ADA) 14.58 IU/L. Tumor markers in the pleural fluid were within normal limits: CEA 0.34 ng/ml, squamous cell carcinoma antigen (SCC) 0.62 ng/ml, and pro-gastrin-releasing peptide (pro-GRP) 23.9 pg/ml. Two consecutive cytological examinations of the pleural fluid showed no evidence of malignant cells.

**Figure 2:**
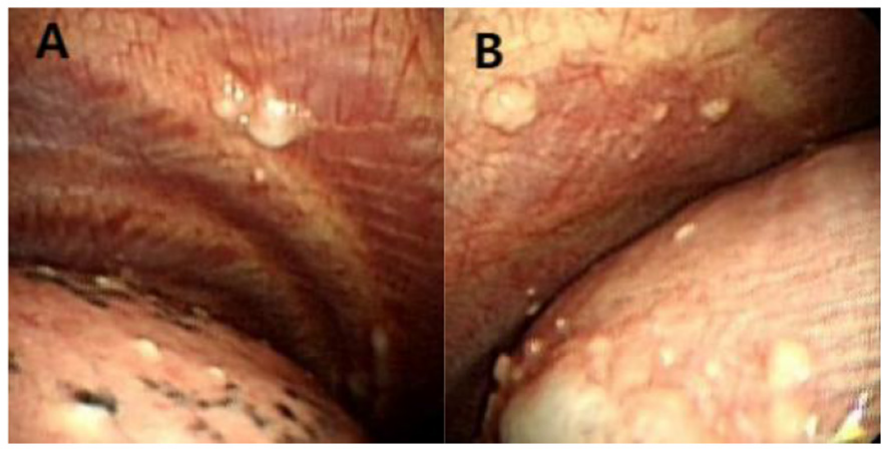
**(A and B)**Thoracoscopy demonstrated amounts of white nodules on the parietal pleura, visceral pleura, and diaphragm.

**Figure 3:**
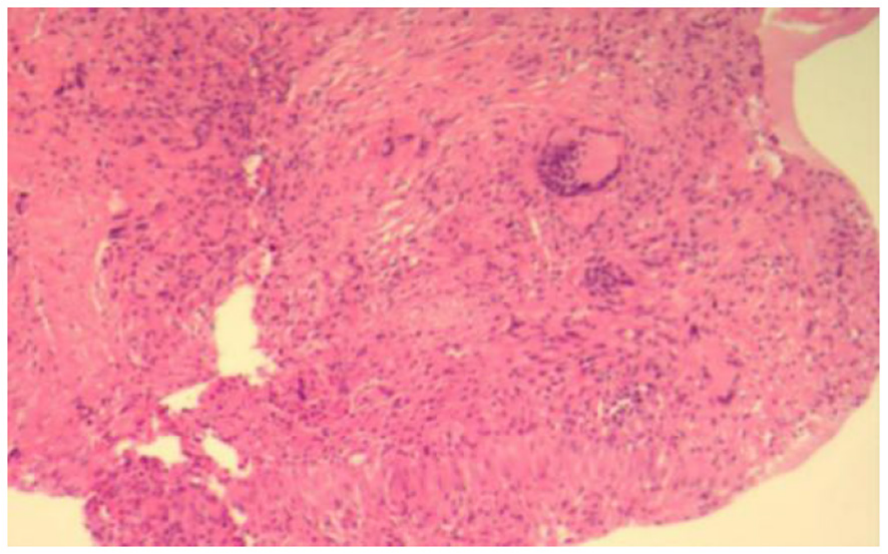
Histologic image of non-caseating granulomas (H&E stain, 10x).

To clarify the etiology of the pleural effusion, video-assisted thoracoscopic surgery (VATS) was performed. Intraoperative findings included numerous white nodules on the parietal pleura, visceral pleura, and diaphragm (Figure 2). Pleural adhesions were minimal, which is inconsistent with the typical thoracoscopic manifestations of malignant or tuberculous pleural effusion. Histopathological examination of the parietal pleural biopsy specimen showed non-caseating granulomas, and acid-fast bacillus (AFB) and fungal stains were negative (Figure 3).

**Figure 4:**
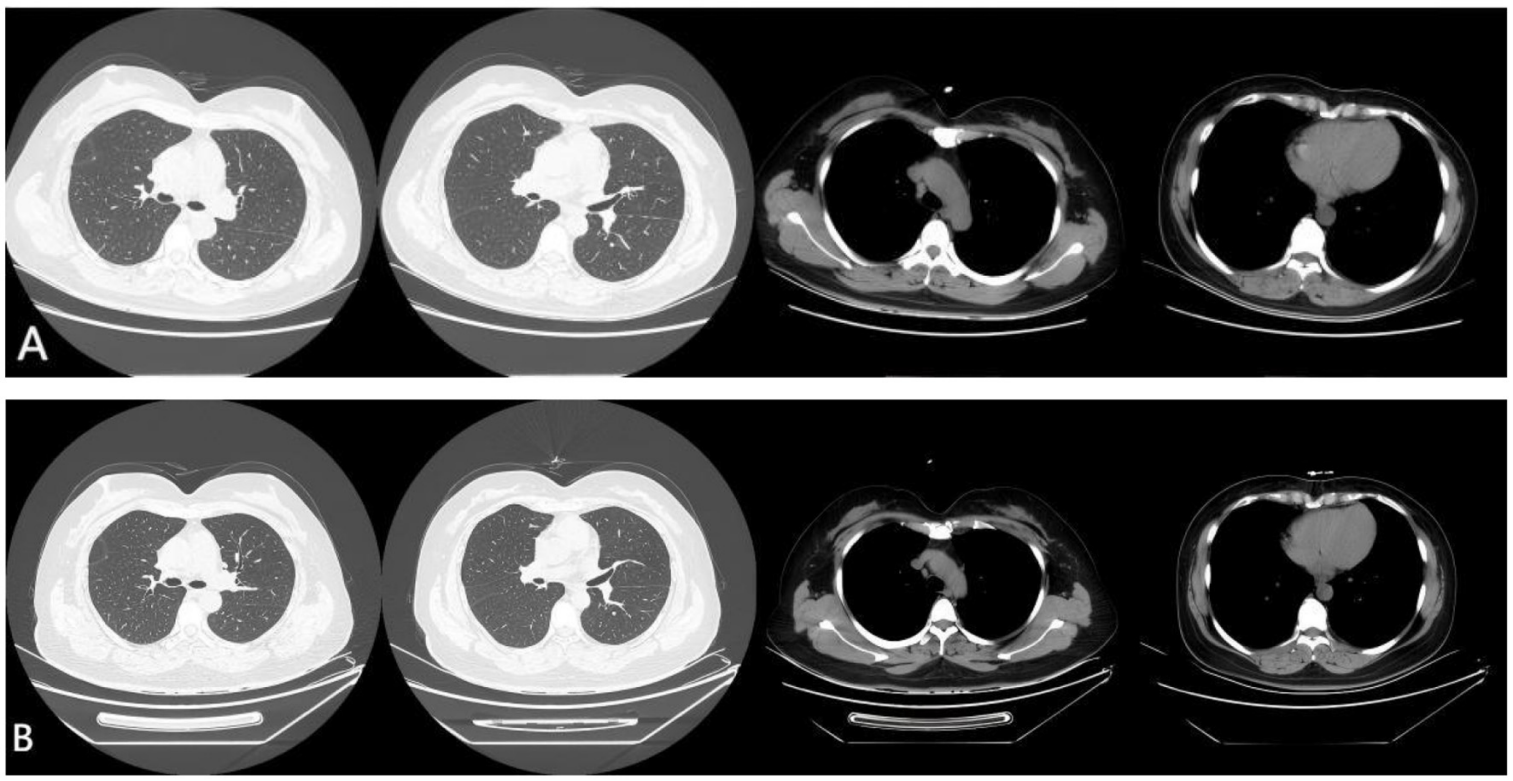
Follow-up chest CT showed a complete withdrawal **(A)** and no recurrence of the pulmonary nodules, subpleural nodules and bilateral pleural effusion **(B)**.

Based on the aforementioned findings, sarcoidosis was diagnosed. Through relevant examinations, extra-thoracic sarcoidosis, such as ocular sarcoidosis, hepatic sarcoidosis and cardiac sarcoidosis, had been excluded. The patient was initiated on prednisolone 40 mg/day according to Delphi consensus recommendations, with a subsequent tapering dosage regimen of 10 mg every 2 weeks (8). Her symptoms of dry cough and dyspnea resolved gradually after treatment initiation. A follow-up chest CT performed 8 weeks later demonstrated regression of pulmonary nodules, subpleural nodules and mediastinal lymphadenopathy, with no recurrence of pleural effusion (Figure 4A). Therefore, the patient discontinued prednisolone therapy. She underwent a follow-up chest CT 8 weeks later, which showed no recurrence of the pulmonary nodules, subpleural nodules and pleural effusion (Figure 4B).

## Discussion

Pleural manifestations of sarcoidosis are generally rare in clinical practice, and the overall prevalence is difficult to assess due to incomplete or delayed reporting. Although most patients with sarcoidosis are diagnosed in early adulthood, those with pleural sarcoidosis tend to have an older age at onset, typically ranging from 30 to 50 years (9). In this case, the patient developed pleural sarcoidosis at 44 years of age. The main patterns of pleural involvement in sarcoidosis include pleural effusion, pleural nodules, pleural thickening, and pneumothorax. It has been reported that sarcoidosis-related pleural effusion is mainly unilateral, with a slight predilection for the right side over the left, though bilateral effusion has also been documented (4, 10, 11). Most sarcoidosis-related pleural effusions are small to moderate in volume, while massive effusions are rarely described (12–14). Although rare cases of eosinophilic effusion, hemothorax, and chylothorax have been reported, the typical cellular feature of sarcoidosis-induced pleural effusion is an exudate with lymphocytic predominance (9, 15–17). The mechanism underlying pleural effusion formation may be similar to that of other infiltrative diseases, and increased capillary permeability caused by pleural involvement is considered one of the pathogenic factors of sarcoidosis-associated pleural effusion. In the present case, the patient presented with pleural sarcoidosis and bilateral lymphocytic exudative pleural effusion, which is consistent with the aforementioned findings.

The diagnosis of sarcoidosis involving the pleura is based on histopathological evidence of non-caseating granulomas, coupled with the exclusion of other granulomatous diseases such as granulomatosis with polyangiitis, tuberculosis, and fungal infections (18, 19). The application of computed tomography (CT) has enhanced the understanding of this unusual site of sarcoidosis involvement, yet it lacks histopathological confirmation. Although thoracentesis or closed pleural biopsy also assist in diagnosis, it is challenging for clinicians to obtain accurate pathological evidence. Medical thoracoscopy provides physicians with crucial clues to identify the cause of pleural effusion in such cases (20). This procedure allows direct exploration and evaluation of the pleural cavity, as well as the acquisition of sufficient tissue samples with minimal invasiveness. In the present study, sarcoidosis-related pleural lesions involving the parietal, visceral, and diaphragmatic pleura were identified and pathologically confirmed with the aid of thoracoscopy. Specifically, pleural biopsy was performed on the patient, and non-caseating granulomas were observed histopathologically, confirming the presence of sarcoidosis-related pleural involvement.

Sarcoidosis-related pleural effusions may resolve spontaneously or require immunosuppressive therapy for resolution. (4, 21). For symptomatic or recurrent patients with sarcoidosis and pleural involvement, oral corticosteroids are the mainstay of sarcoidosis treatment and the most commonly used first-line clinical therapy (22–24). Corticosteroids inhibit granuloma formation, thereby exerting significant efficacy against active manifestations of sarcoidosis. The time to resolution of sarcoidosis-related pleural effusions varies, but most cases improve within 2–3 months (10, 19). In this case, corticosteroid therapy led to significant improvement of both pleurisy and parenchymal infiltrates. The patient responded well to oral corticosteroid treatment, with marked improvements in both clinical symptoms and chest radiological findings.

## Conclusion

In summary, this case presents a rare form of pleural involvement in sarcoidosis manifesting as pleural effusion. The definitive diagnosis of sarcoidosis with pleural involvement relies on histopathological confirmation of non-caseating granulomas in pleural tissue, coupled with the exclusion of other potential etiologies. Thoracoscopy plays a pivotal role in the diagnosis of pleural sarcoidosis, and corticosteroid therapy has demonstrated favorable efficacy in treating pleural sarcoidosis.

## Data Availability

The raw data supporting the conclusions of this article will be made available by the authors, without undue reservation.
